# Dynamics of odor sampling strategies in mice

**DOI:** 10.1371/journal.pone.0237756

**Published:** 2020-08-14

**Authors:** Johannes Reisert, Glen J. Golden, Michele Dibattista, Alan Gelperin

**Affiliations:** 1 Monell Chemical Senses Center, Philadelphia, PA, United States of America; 2 Department of Basic Medical Sciences, Neuroscience and Sensory Organs, University of Bari “A. Moro”, Bari, Italy; 3 Department of Neuroscience, Princeton University, Princeton, NJ, United States of America; Newcastle University, UNITED KINGDOM

## Abstract

Mammalian olfactory receptor neurons in the nasal cavity are stimulated by odorants carried by the inhaled air and their activation is therefore tied to and driven by the breathing or sniffing frequency. Sniffing frequency can be deliberately modulated to alter how odorants stimulate olfactory receptor neurons, giving the animal control over the frequency of odorant exposure to potentially aid odorant detection and discrimination. We monitored sniffing behaviors and odorant discrimination ability of freely-moving mice while they sampled either decreasing concentrations of target odorants or sampled a fixed target odorant concentration in the presence of a background of increasing odorant concentrations, using a Go-NoGo behavioral paradigm. This allowed us to ask how mice alter their odorant sampling duration and sampling (sniffing) frequency depending on the demands of the task and its difficulty. Mice showed an anticipatory increase in sniffing rate prior to odorant exposure and chose to sample for longer durations when exposed to odorants as compared to the solvent control odorant. Similarly, mice also took more odorant sampling sniffs when exposed to target odorants compared to the solvent control odorant. In general, odorant sampling strategies became more similar the more difficult the task was, e.g. the lower the target odorant concentration or the lower the target odorant contrast relative to the background odorant, suggesting that sniffing patterns are not preset, but are dynamically modulated by the particular task and its difficulty.

## Introduction

It is now clear that the dynamics of odor sampling during the first few sniffs taken by rodents during an odor identification task can provide enough information to allow odor identification and discrimination [1–3]. The importance of odor sampling by active sniffing for olfactory perception by humans and rodents has been known for more than three decades [4–8]. Active sniffing or odor sampling strategies also dramatically alter synaptic interactions in the olfactory bulb, the first central synaptic processing site for odor-elicited sensory input [9,10]. The temporal dynamics of olfactory receptor neuron (ORN) responses during odor sampling is a critical determinant of the information available to the olfactory bulb [11–13] or its analog, the antennal lobe in insects [14].

Sniffing is one of a set of orofacial motor behaviors that includes, in addition to sniffing, breathing, whisker movements, nares movements and head positioning [15]. Circuits in the ventral medulla of the brainstem are likely to provide the coupled rhythmic motor control for the essential coordination of sniffing and whisking [15–18]. This renewed interest in the coordination of rodent orofacial motor behaviors has led to the development of novel technologies for measuring rodent breathing and sniffing [19,20], which are complementary to the method described here. Recent work has further emphasized the importance of sniff dynamics for odor identity coding and discrimination [1,21–23], augmented by new analytical approaches to analyzing olfactory network dynamics, particularly the use of time warping [24,25].

The experiments described here use a combination of olfactory psychophysics and measurements of sniffing in a freely moving mouse [19,26] during odor-guided behavior to address how sniff-driven odor-elicited activation patterns in ORNs drive behavioral outputs.

## Materials and methods

All surgical procedures and handling of mice were approved by the Institutional Animal Care and Use Committee of the Monell Chemical Senses Center. Only male 129S6 mice were used in this study. Mice were water-deprived prior to experiments and received small water rewards during the Go/NoGo behavioral experiments. Additional water was supplemented such that mice maintained at least 85% of their body weight.

### Acquiring breathing signals

To monitor breathing and sniffing, telemetric thoracic breathing sensors [PhysioTel TA11PA-C10, Data Sciences International (DSI), St. Paul, USA)] were implanted as described previously [26–28]. In short, mice were anesthetized with isoflurane and depth of anesthesia was tested with a toe pinch. Following surgery, mice were injected with an analgesic (0.5–2.0 mg/kg Buprenorphine s.c.) to alleviate potential postoperative pain. The sensor catheter of the TA11PA-C10 was inserted into a small incision in the serosal layer of the esophagus and tunneled cranially past the diaphragm into the thoracic cavity. The catheter was secured at the point of entry and the body of the transmitter was sutured to the abdominal wall. The pleural pressure signal was sensed by a receiver platform (RPC-1) and recorded using a commercial telemetry system (Matrix 3643, DSI). Data were sampled at 500 Hz and filtered at DC– 100 Hz and recorded continuously during the experiments. Subsequently, the data were analyzed offline using custom written MATLAB software. First, it was band-pass filtered at 3–15 Hz to remove baseline drifts and high frequency noise and then the timings of inhalation and exhalation peaks were extracted to calculate the inter-sniff intervals and the breathing frequencies. Following the conclusion of the study, mice were euthanized with CO_2_ followed by cervical dislocation.

### Odorant-driven behavioral experiments

The methods used for training mice are adapted from those described in [29]. Other modifications are listed below. An olfactometer (Knosys, Lutz, USA) based on the original Bodyak & Slotnick design [30] was modified to have two ports, one to sample odorants and a second separate port to obtain water rewards [26]. When the mouse’s nose broke an IR beam that crossed the entrance to the odor port, a new trial was initiated and an odorant from one of eight odorant vials was delivered to the odor port after a 0.5 s delay. The odorant was delivered continuously until the mouse withdrew from the odor port. Selection of the odor reservoir and its behavioral meaning (Go, NoGo), and measurement of time stamps (odor port in/out, water port in/out) were measured and controlled using ABET software (Lafayette Instruments, Lafayette, USA). The odorant used was 1-propanol at vol/vol dilution as indicated and dissolved in filtered mineral oil.

Mice were trained to perform Go/NoGo tasks, where delivery of an odorant was associated with a water reward, while no odor signaled the lack of reward. To encourage mice to stay in the odor port for longer durations such that they could take advantage of longer sampling times to improve their accuracy [2,31,32], mice were trained to stay in the odor port for up to a total of 1.5 s. Once the task was learned, mice were trained on the Go/NoGo paradigm at a propanol concentration of 10^−4^ vol/vol dilution with no restrictions on how long they stayed in the odor port.

Mice performed two types of odorant-driven experiments: Dose Response and Adaptation experiments.

### Dose response experiments

The first experiment determined the dependency of sniffing behavior, sampling duration and odorant identification accuracy on progressively lower odorant concentrations. Experiments were run in blocks of 20 trials, of which 10 trials were water-rewarded S+ (but see below) and 10 trials were unrewarded S- trials, respectively. S+ and S- trials were presented in random order with the restriction that no more than four S+ or three S- trials occurred in a row. For S+ trials, we adopted a 70/30 reward schedule, (only 7 out of 10 trails were rewarded with water) to accustom mice to the fact that, particularly during difficult trials, perceived S+ events will not always be rewarded. In preliminary experiments we noticed that mice could use cues from other signals (e.g. sound of switching valves, vibrations, slight changes in air flow associated with different valves) than odorant concentration to determine their behavioral choice, particularly during more difficult tasks with low odorant concentrations. Thus, we adopted the following protocol to force the mice to use perception of the odorant as the cue for their behavioral decision. The eight available odorant reservoirs were assigned randomly each day as follows. Two vials contained propanol at 10^−4^ and two contained control mineral oil (MO) (termed collectively the “concordance vials”). Two vials were the test odorant concentration for that day and its MO control, while the remaining two vials were, once again, 10^−4^ propanol and MO. The latter two were termed the test* vials. For each test propanol concentration, three blocks of test trials were recorded. Subsequently, data derived from the three blocks was analyzed and averaged for each mouse.

At the beginning of each experiment, mice had to conduct three consecutive blocks at 10^−4^ propanol concentration using the four concordance vials at 85% correct or better. This was done to ascertain that mice still performed the behavioral test appropriately. Thereafter they would advance to a test block at a lower concentration (e.g. 10^−5^ using the test vials). Once the test block was completed mice were again tested at blocks at 10^−4^ until they reached an accuracy of >85% before they could run another test block. This ensured that if mice showed very low accuracy during the test block at lower odorant concentrations, they still used the odorant as the cue and did not choose their behavior randomly or just went to the water port on each trial independent of the odorant cue. Typically, three test blocks were performed each day. Once completed, mice were finally tested on the test* vials with a new set of valves they had not encountered before that day to ensure that they, indeed, used the odorant as the behavioral signal and not signals associated with the operation of the olfactometer. When mice performed at <85% correct during the test* block, it was assumed that mice used cues other than the odorant and the experiments for that day were discarded.

### Adaptation experiments

In a second set of experiments, we investigated the effect of background odorant concentration and the consequent olfactory adaptation on odor recognition performance using an approach similar to Kelliher et al. [33]. Mice were always tested at a propanol concentration of 10^−4^, two S+ (10^−4^) and two S- (MO) vials were assigned randomly each day. To odorant adapt mice, the behavioral chamber was filled with a constant stream of odorized air coming from an odor reservoir that was filled with increasing propanol concentrations as indicated below. For the first block of an experiment, the chamber was filled with clean air, and if mice reached at least 85% accuracy, air from a vial containing MO was introduced into the behavioral chamber for the next block. If mice reached at least 85% accuracy, the next block was performed at a given background propanol odorant concentration. Only if mice reached at least 85% accuracy in following blocks of MO as the background, was the block included in the analysis. For each background propanol concentration, typically 5–6 blocks of test trials were recorded and subsequently analyzed and averaged for each mouse. For mineral oil as the background odorant concentration, 10–15 blocks were recorded.

Mouse responses were categorized as follows:

Hits = mouse gets a water reward to the S^+^ odor, FA = False Alarm when the mouse seeks a water reward to the S^-^ odor, CR = Correct Rejection when the mouse does not seek water to the S^-^ odor, Miss = failure to seek water reward to S^+^ odor.

Misses were excluded from data analysis when less than 0.75% of trials were scored as a Miss for mice in the adaptation experiments.

Data analysis was performed with R [R Core Team (2018). R: A Language and environment for statistical computing] for the mixed model ANOVA where the fixed effects were the concentration of the odorant and the mouse behavior and subjects as random effect (intercepts varying across mice). Jamovi [The jamovi project (2019), https://www.jamovi.org] was used for repeated measure AVONA. Post-hoc comparisons were performed as stated in the figure legends.

## Results

**Fig 1:**
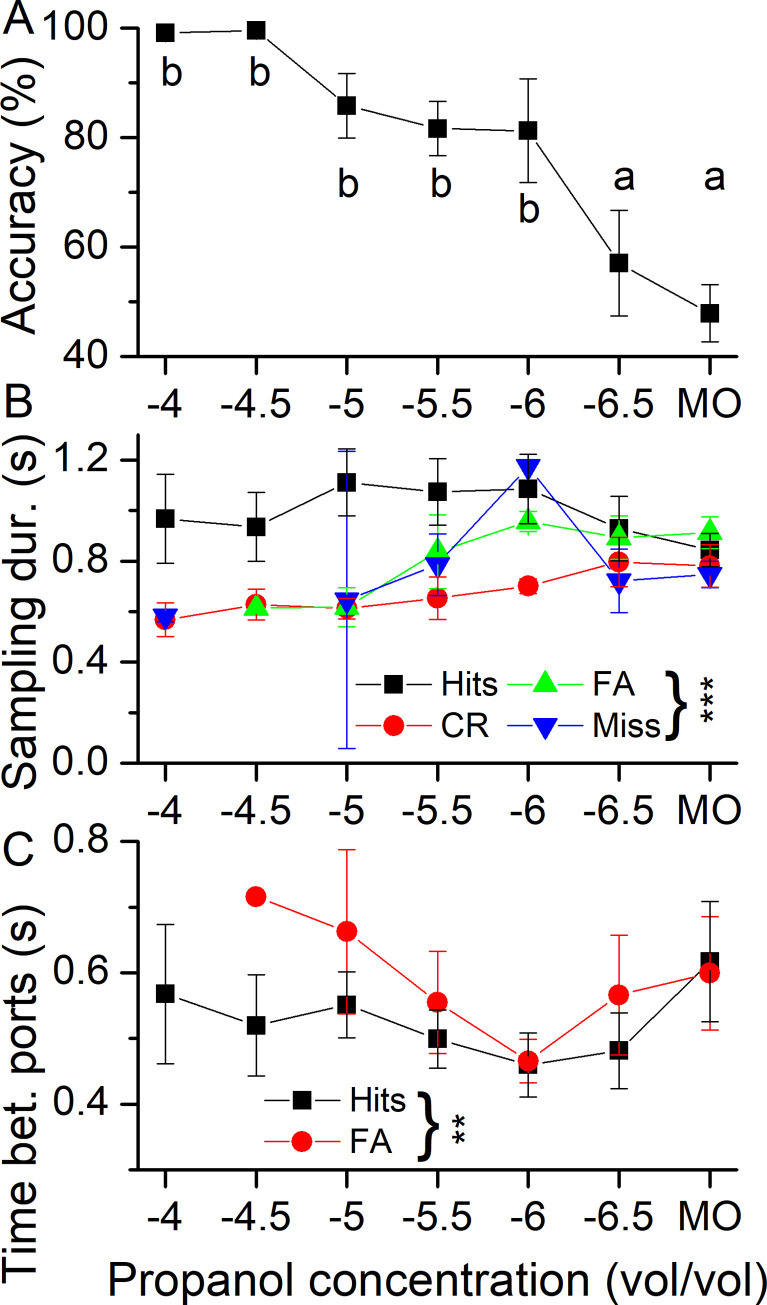
Accuracy and sampling behavior in a Go/NoGo odor-guided experiment. **A** Mice were tested with increasingly lower propanol concentrations against the carrier mineral oil (MO) to determine their accuracy to detect the presence of the odorant. Accuracy across odorant concentrations was assessed by a repeated measures ANOVA (F(6,18) = 17.1, p < 0.001) followed by post-hoc Tukey. Same letters indicate the absence of significance. **B** The odor sampling duration as a function of both the odorant concentration and the four different behavioral outcomes: Hits, Correct Rejections (CRs), False Alarms (FAs) and Miss. Sampling duration changes significantly depending on the mouse behavior (mouse behavior main effect in mixed model ANOVA F = 18.01, p < 0.0001). Post-hoc comparisons showed that CRs are significantly different from the Hits (Post-hoc with Bonferroni correction p = 2.02e^-10^). **C** The time mice spent between the odor and the water port following their behavioral decision. In all graphs, average (± SEM) of four mice is shown, each tested for three blocks at each concentration. The x-axis displays the log 10 of the odorant concentration.

The first goal of our analysis was to ensure that we could obtain consistent and quantitative measures of odor-guided choice behavior of mice challenged with identification of increasingly diluted propanol concentrations when motivated by small (3 μL) water rewards after water deprivation. The data in Fig 1A, obtained from four mice, demonstrate that this is the case. The mice showed near 100% accuracy at a propanol concentration of 10^−4^% (vol/vol) concentration, which began to decline with declining odorant concentration and showed accuracy near chance, equivalent to that elicited by the odor of the mineral oil (MO) vehicle at 10^−6.5^%. The odorant sampling duration, the time mice chose to sample the target odorant, stayed relatively constant for Hits over this same concentration range (Fig 1B), while the sampling duration for correct rejections (CRs) was much shorter at easier discrimination tasks, but became similar to Hits at low odorant concentrations. False alarms (FAs) and Misses showed a more variable dependency on odorant concentration. The transit time when the mouse moved from the odor port to the water port (time between ports) for Hits and False Alarms (FAs), the two events when such a transition can occur, stayed constant over the tested concentration range (Fig 1C) but during FAs, mice took significantly longer to transition to the odor port. This suggests different speeds to execute the mouse’s behavioral decision. For the propanol concentration of 10^−4^, no FAs were observed for the entire dataset of four mice and no Misses occurred at 10^−4.5^.

**Fig 2:**
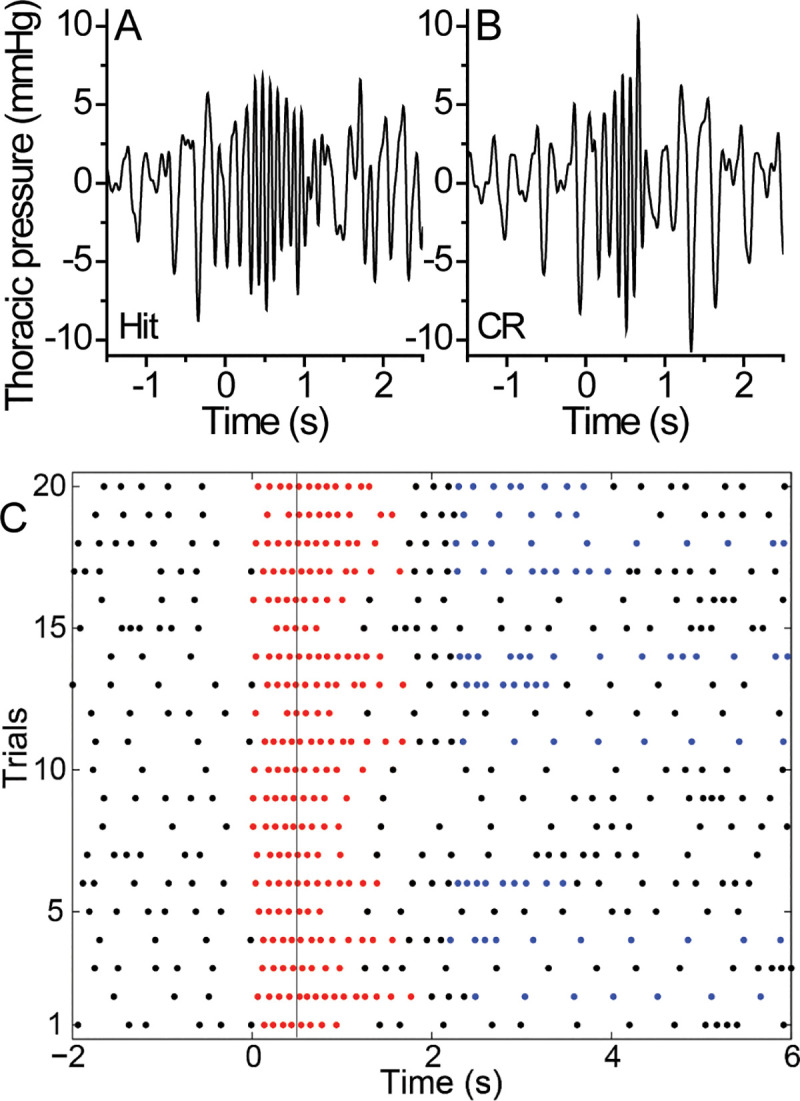
Recordings of thoracic pressure changes during odorant sampling. The breathing pattern during (**A**) Hit and (**B**) a correct rejection (CR) trial when exposed to 10^−4^ propanol concentration. Time 0 is the onset of the nose poke into the odor port. The odorant was delivered at 0.5 s. **C** Display of 20 trials of a 10^−4^ propanol concentration block. Each dot represents an inhalation, a red dot means the mouse’s nose is in the odor port, a blue dot means the mouse’s snout is in the water port and a black dot means that the mouse’s nose is in neither port. The grey line is the time of odorant delivery onset. Trials lacking blue dots (meaning the mouse did not enter the water port) are, for this chosen block, all CRs as no false alarms (FAs) occurred.

Additionally, we measured breathing and sniffing frequencies in the unrestrained mouse while it made a large series of odor-guided decisions in the computer-controlled olfactometer. Examples of the filtered data (see Materials and methods) derived from the wirelessly-recorded thoracic pressure sensor are shown in Fig 2A and 2B. Fig 2A shows the thoracic pressure fluctuations corresponding to sniffing prior to, in and following withdrawal from the odor port during a Hit of 10^−4^ propanol while Fig 2B shows the sniffing pattern shown for a single trial of a CR of the MO vehicle. Mice entered the odor port at t = 0 s and began to increase their sniffing frequency even prior to the time of target odorant delivery or the control odorant delivery at 0.5 s in both types of trials. Fig 2C shows a raster plot of 20 trials and displays the peaks of each sniffing transient marked by a dot for a single block (10 Hits and 10 CRs). It shows the consistency of the onset of sniffing at the onset of the nose poke and the variability of the offset of sniffing after odor delivery accompanied by a variable latency of termination of the nose poke.

**Fig 3:**
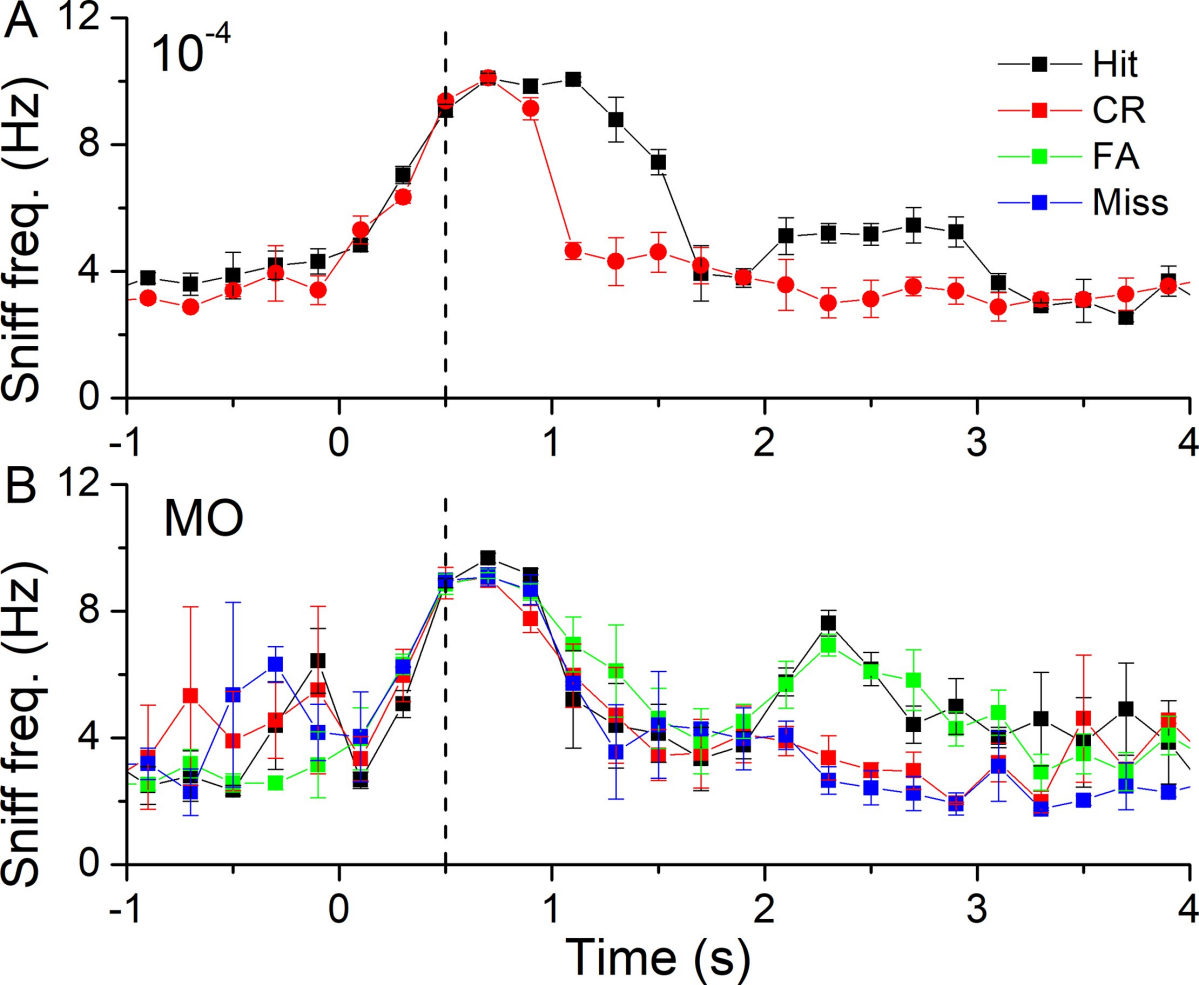
Sniff patterns during odorant sampling. The sniff frequency of a mouse when tested with (**A**) a 10^−4^ propanol concentration or (**B**) when the S+ odorant was mineral oil (MO). Sniff frequencies were binned in 0.2 s bins according to their behavioral outcome and averaged (mean ± SEM) across trials of one block of 20 trials. Vertical dashed line indicates odorant onset.

We analyzed in detail the differences in sniffing frequency between trials and between different odorant concentrations. Fig 3 shows this analysis for 10^−4^ propanol versus the sniffing frequency of trials when the control odor of the vehicle (MO) was delivered. For each block and a given behavioral outcome, the sniffing frequency was binned into 200 ms time windows and averaged across all relevant trials in the block. As shown in Fig 3A for 10^−4^ propanol the initial rise of and the peak sniffing frequency is the same for Hits and CRs but sniffing drops to baseline after 0.5 s of odorant sampling for CRs while sniffing frequency remains elevated for three times as long during Hit trials. The sniffing frequency changes are similar for all four behavioral outcomes when responding to the odor of the vehicle, MO (Fig 3B). Often, trials that involved the mouse entering the water port [Hits and False Alarms (FA)], triggered an increase in sniff frequency at 2–3 s, which did not occur during CRs and Misses.

**Fig 4:**
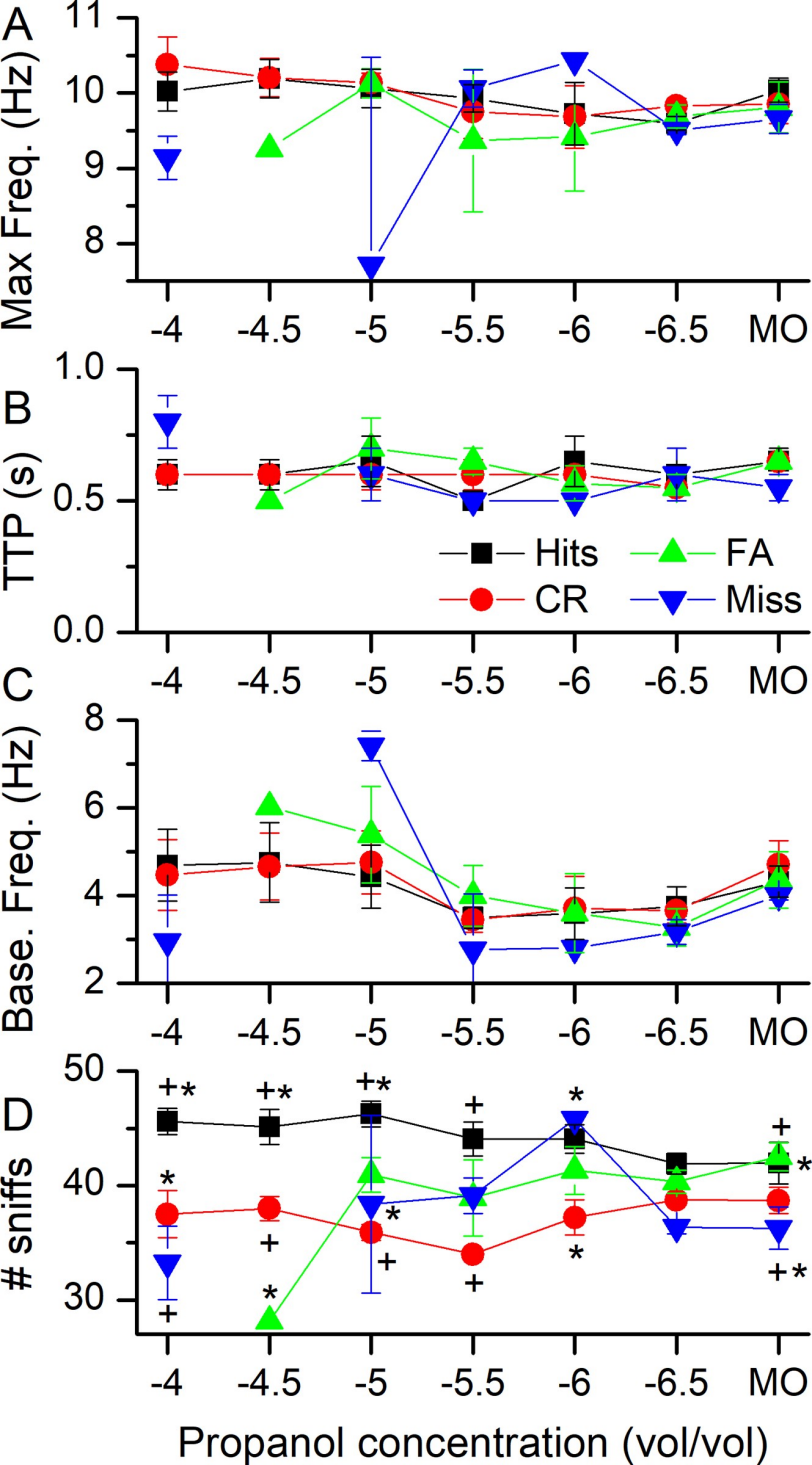
Analysis of multiple parameters of sniffing behavior during the four types of responses to odorant sampling. **A** The maximal sniff frequency during odorant sampling. **B** The time to reach maximal sniff frequency calculated from the odorant onset. TTP = time to peak. **C** Baseline sniff frequency averaged across the sniffing event from -1 to 0 s. **D** The total number of sniffs taken during the five 200 ms bins centered on 0.5 to 1.3 s. Mixed model ANOVA with interaction term shows mouse behavior and concentration being significant (F = 2.4, p = 0.008), symbols at each concentration indicate statistical significance between different behavioral output at a given odorant concentration. All data are mean ± SEM for four mice.

We next analyzed four different parameters that characterize the sniffing responses to odorant delivery in the olfactometer, namely, the maximum sniffing frequency, the time from the onset of the nose poke to the occurrence of the maximum sniffing frequency (TTP), the baseline sniffing frequency prior to the initiation of the nose poke, and the number of sniffs that occurred during the 1 s after odorant delivery. These data, representing averages from four mice, are shown in Fig 4. The maximum frequency (Fig 4A), the time to peak frequency (Fig 4B) and baseline sniffing frequency (Fig 4C) show similar, stable, patterns across the four types of mouse responses for Hits and CRs, with indications of more variability for FAs and Misses. The number of sniffs taken in response to odor delivery (Fig 4D) shows a marked elevation for trials scored as a Hit versus trials scored as CRs, particularly at the higher propanol concentrations, which is due to the longer elevated sniffing frequency observed during Hit trials. For high odorant concentrations, the number of sniffs is different between Hits and FAs, until at 10^−6.5^ and MO, the number of sniffs becomes similar. The numbers of sniffs taken for FAs are different only when compared to Hits at the high odorant concentrations of 10^−4.5^ (no FAs were observed at 10^−4^) and occupied a middle ground between Hits and CRs, not being statistically different from either Hits or CRs. This suggests that, while the actual presence of an odorant can determine the number of sniffs taken, the sniff pattern can also influence or interfere with the behavioral outcome (FAs).

**Fig 5:**
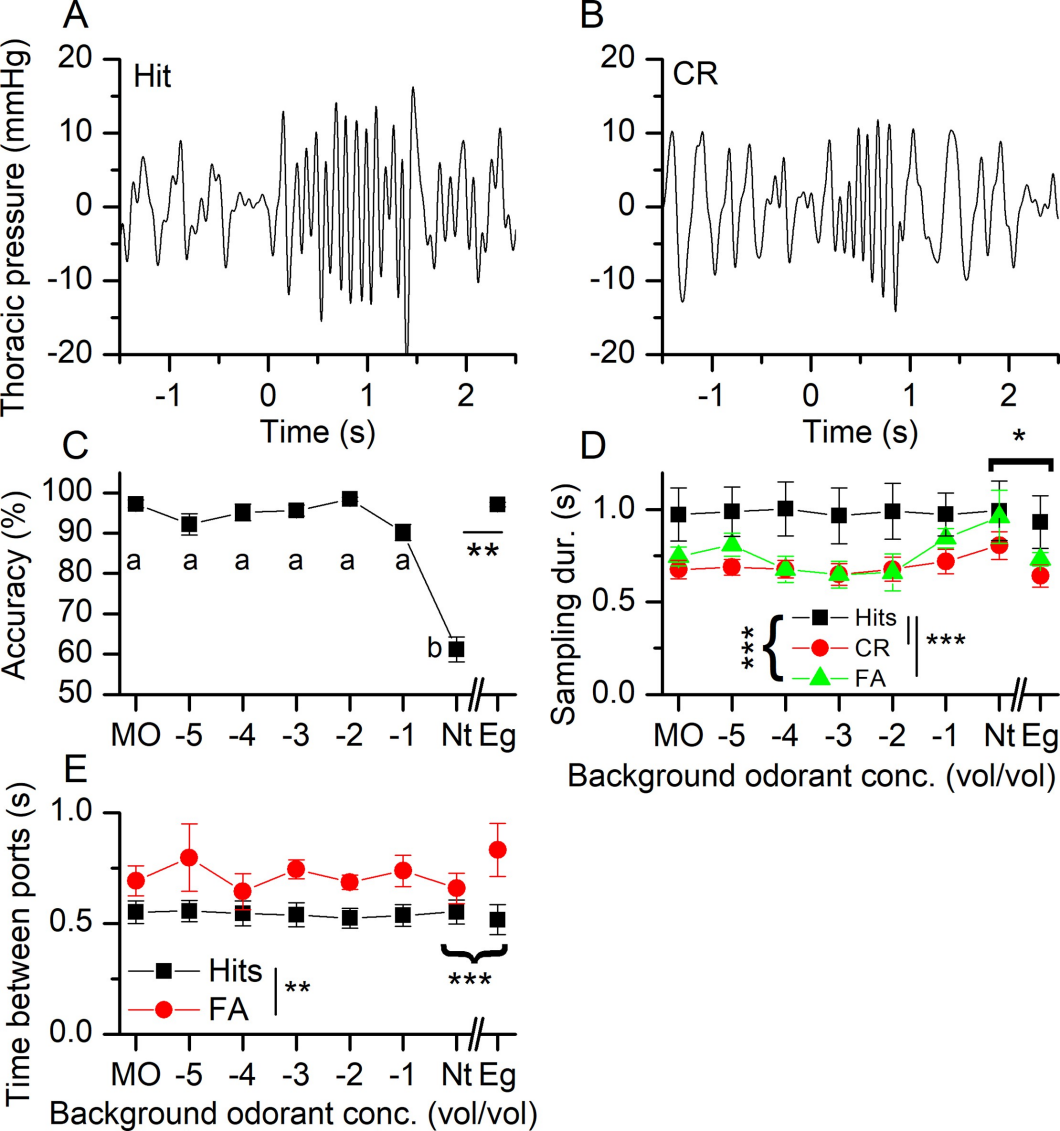
The effect of background odorant concentration on olfactory acuity. Mice were tested to distinguish 10^−4^ propanol from the carrier mineral oil (MO) in the presence of increasing background concentrations of propanol up to undiluted (neat, Nt) propanol and also undiluted eugenol (Eg). The breathing pattern during (**A**) Hit and (**B**) a correct rejection (CR) trial when exposed to 10^−4^ propanol concentration in the odor port in the background of MO. Time 0 is the onset of the nose poke into the odor port. The odorant was delivered at 0.5 s. **C** Response accuracy, same letters indicate the absence of significance, repeated measures ANOVA (F(6,18) = 45.2 p = 0.001) followed by post-hoc Tukey. Neat (Nt) propanol and eugenol (Eg) were significantly different (paired t-test, p = 0.0018). **D** The odorant sampling duration for Hits, CR and FA. Mixed Model ANOVA revealed mouse behavior as main effects with F = 1.6, p = 1.86e^-09^. Hits were different from CRs and FAs (Post-hoc with Tukey’s correction p < 0.001 respectively). Comparison of Nt vs Eg showed both behavioral outcome and stimulus as main effects, F = 4.7, p = 0.026 and F(1,15) = 5.6, p = 0.031 respectively. **E** The time taken by mice to transition from the odor to the water port for Hits and FA (statistically different, Mixed model ANOVA, F = 41, p = 1.7 e^-7^). For Nt vs Eg, the interaction term is significant (F = 0.2 p = 0.02) and post-hoc analysis reveals that Eg Hits are different from Eg FA (p = 0.0002) while Nt was not different between the two (p = 0.0771). Only for FAs were Nt and Eg different (p = 0.01). Data are mean ± SEM of four mice. “{“indicate comparison across behavioral outcomes, while “[“indicated comparison across odor stimuli.

We next performed an analysis of the accuracy of target odorant identification (propanol) in the presence of various concentrations of the target odorant as a background odorant pervading the behavioral chamber of the olfactometer. This analysis investigated the performance of mice with the aim of revealing differences in olfactory recognition ability in a challenging version of the target odorant identification task when the behavioral chamber was permeated with increasing background concentrations of the target odorant. The odorant concentration delivered in the odor port was always 10^−4^. Fig 5A & 5B show sniff traces for a Hit and a CR respectively in the presence of MO as the control background odor. Again, sniff frequency increases as the mouse enters the odor port prior to the propanol stimulation at 0.5 s. During a Hit, the sniff frequency stayed elevated for around 1.5 s, while during a CR trial, sniff frequency declined quickly within 1 s. For background concentrations up to 10^−1^ mice performed at very high accuracy. Only at the highest background concentration of propanol did the accuracy of mice drop to near chance (Fig 5C). It might be surprising that performance only declined at very high background odorant concentrations, nominally at concentrations that are much higher than the odorant concentration to be detected in the odor port. Note that the stated odorant concentrations are the vol/vol dilution in the vials that supply the odor port and the behavioral chamber. In the vials that supply the odor port, odorants from the odorant solution will equilibrate in the headspace of the vial until used to supply the odor port. For the vial that supplies the behavioral chamber, air continuously flows through the vial into the chamber, thus potentially reaching lower concentrations compared to an equilibrated situation. Note also that the use of full strength undiluted propanol (neat, Nt) as the background odor severely depressed the identification accuracy of the mice, whereas the use of undiluted eugenol (Eg) did not depress the odorant identification accuracy. This suggests that it is not the high odorant concentration per se, but rather specific adaptation to propanol, or alternatively, that the concentration contrast between the background and the test odorant concentration is not perceived by the adapted ORNs. Cross-adaptation to eugenol does not affect the accuracy to propanol.

We used our ability to measure multiple quantitative parameters of mouse odor sampling behavior to look more closely for differences in odor sampling behavior, particularly the effects of background odors matching the target odor on odor sampling duration. Fig 5D compares the sampling durations for Hits, CRs and FAs. Misses were omitted from the analysis as mentioned in the Materials and methods. Mice sample longer during Hits when compared to both CRs and FAs, but CRs and FAs were not different from each other. Both for Hits and CRs the sampling duration was quite stable irrespective of the background concentration and also the declining accuracy at higher background odorant concentrations (see Fig 5A), while for FAs sampling duration tended to fluctuate more. The comparison between Nt and Eg revealed that sampling duration overall was shorter for Eg compared to Nt.

Additional analysis of the quantitative parameters of odor sampling behavior focused on a comparison of the time taken to transition between the odor port and the water port, as shown in Fig 5E. Only the Hits and FA trials involved a sequential visit first to the odor port and then the water port so only data from these two categories of trials are compared. As in the previous data shown above, the odor identification task was made more difficult by flooding the olfactometer behavioral chamber with either increasing concentrations of the target odor (propanol) or an unrelated odor, eugenol (Eg). With this behavioral task, the transition times between the odor and the water ports were quite stable across the tested propanol concentration for both Hits and FAs, but mice took significantly longer to transition during FAs. For the comparison between Nt and Eg, the time between ports for the behavioral outcomes were different from each other.

**Fig 6:**
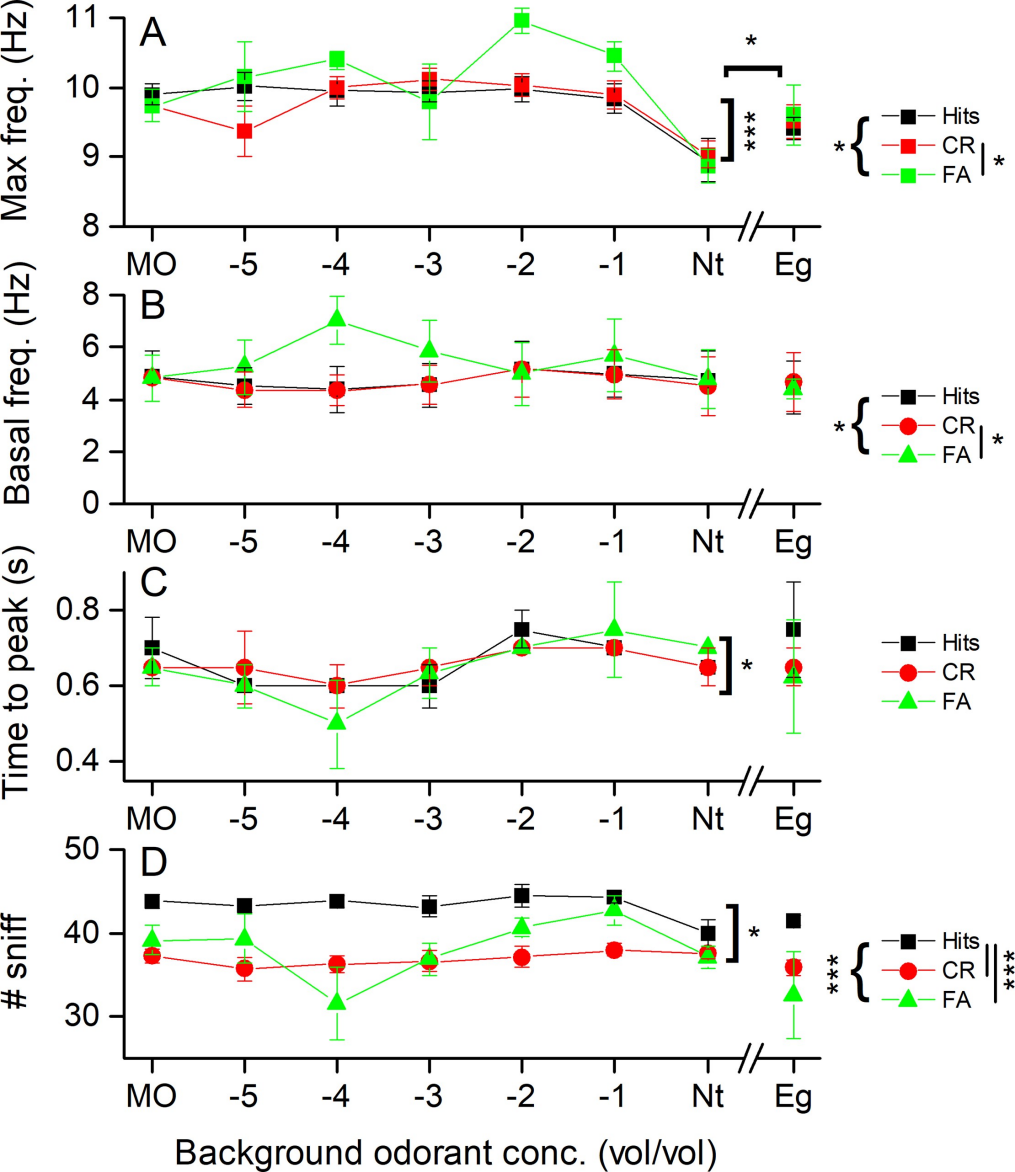
Analysis of sniffing behavior depending on the odorant background concentration. **A** The maximal sniff frequency during odorant sampling for Hits, CR and FA. Maximal frequency is significantly different for propanol concentrations (Mixed model ANOVA, concentration main effect F = 12.4, p = 6 e^-9^) and behavioral outcome across Hits, CR and FA (mouse behavior main effect F = 4.16 p = 0.020). Post-hoc comparisons showed that maximal frequency is significantly different between CR and FA (p = 0.023). Nt vs Eg has the stimulus as the main effect (F = 8, p = 0.012). **B** Basal sniff frequency during the 1 s prior to entering the odor port. Basal frequency of FA is statistically different (Mixed model ANOVA mouse behavior main effect F = 4.1 p = 0.022. Post-hoc Tukey’s did statistical difference between FA and CR (p = 0.03). **C** The time to reach maximal sniff frequency following entry into the odor port (Mixed model ANOVA main effect background odorant concentration, F = 2.6, p = 0.026. **D** The total number of sniffs taken depending on the behavioral outcome. Mixed model ANOVA revealed for the propanol backgrounds that Hits are significantly different from CR and FA (mouse behavior as main effect F = 33.2, p = 2 e^-10^ and background propanol concentration as main effect F(6,60) = 3.0, p = 0.012 and Post-hoc Tukey’s Hits vs CR p < 0.0001 and Hits vs FA p < 0.0001). Nt and Eugenol are not different (Nt and Eg main effect Mixed model ANOVA). All data are the sum of sniffs in the five bins from 0.5 to 1.3 s. Data are mean ± SEM of four mice. “{“indicate comparison across behavioral outcomes, while “[“indicated comparison across odor stimuli. Statistics and significance levels indicated on the right for Hits, CR and FA refer to comparison of the experiments with propanol and MO.

Our analysis of differences in the parameters used to characterize odorant identification behavior in the olfactometer now focused on potential differences between the maximum sniffing frequency, the basal sniffing/breathing frequency and the time from entering the odor port to the time of peak sniffing frequency, when mice were challenged by varying concentrations of a background odorant identical to the target odorant (propanol) or unrelated to the background odorant (eugenol, Eg). These data are shown in Fig 6. The maximal sniff frequency (Fig 6A) decreased at the neat propanol background concentration (with Nt being significantly different from all other concentrations), but showed less of a reduction when eugenol was the background and was significantly increased compared to Nt. These data suggest that it was not the high odorant concentration itself that reduced the maximal sniff frequency, but instead the lack of contrast between the background odorant and the target odorant. The basal sniff frequency (Fig 6B) was very similar for Hits and CRs but interestingly showed more variability for FAs. Overall, the behavioral outcomes were significantly different, and post-hoc tests revealed that CRs were different from FAs (see Fig 6 legend). Time to reach maximal sniff frequency (Fig 6C) varied across background odorant concentration, but was not different across behavioral outcomes.

To further explore the possible differences in odorant identification behavior, we next examined the number of sniffs taken by mice when challenged with a background odorant equivalent to the target odorant propanol and with a background odorant different than the target odorant, e.g., eugenol (Eg). The analysis was first done as a function of the background odorant concentration and as a function of the result of the mouse’s response to the target odorants, i.e., for Hits, CRs and FAs, as shown in Fig 6D. Across the propanol background concentrations, mice took significantly more sniffs when the behavioral outcome was a Hit when compared to both CRs and FAs and numbers of sniffs were also different across the propanol concentrations. Interestingly, during the Nt background for Hits, mice showed a reduction in sniffs taken at this background propanol concentration, although the sampling duration stayed constant at this concentration (see Fig 5D).

## Discussion

Recent work on the minimal odor sampling duration needed by rodents to achieve significant accuracy at odorant identification tasks has indicated that, under some conditions, a single sniff is sufficient to allow odor identification [4,34]. This raises the question as to the normal patterns of sniffing when unrestrained mice are using odor cues to obtain a reinforcement such as a small aliquot of water after application of a significant water-restriction regime prior to behavioral testing. The data presented here provide insight into this issue. Under our test conditions, involving forced odor sampling at an odor delivery port in an olfactometer [29], trained mice initiate an increased rate of breathing, labeled sniffing, upon entering the odor port and prior to the onset of odor delivery as previously also observed in rats [4]. As shown by the comparison of the data in Figs 1B and 3A, mice began to reduce their sniffing frequency even prior to the termination of the odor port nose poke during CRs and reached baseline sniff levels within less than 0.5 s of odorant sampling, which is the time mice sampled for CRs. Also, after 400 ms of odorant or control sampling, sniffing traces began to diverge between Hits and CRs, indicating that, at least at the level of sniffing frequency modulation, the mouse had begun, consciously or unconsciously, to modify its behavior. Given their 10 Hz sniffing frequency at this time point this would equate to an upper boundary of around 4 sniffs prior to making an odor-guided decision. For Hits, mice sampled both longer and also maintained a higher breathing frequency. These mice collected more than the minimal information needed to determine odor identity prior to implementing their behavioral decision during Hits. Why mice maintained a higher sniffing frequency in the presence of odorants during Hits, while they could already detect the lack of odorants during CRs, remains unclear. For harder tasks, e.g. when asked to distinguish MO from MO, both sampling duration and sniff frequency patterns became similar between CRs and Hits. During dose-response experiments (Fig 4), both the maximal sniffing frequency and also the time to reach maximal sniffing frequency stayed constant across tested odorant concentrations, suggesting that these parameters of the sniffing response are relatively stereotyped for this kind of task. The decrease in the number of sniffs taken with increasing difficulty might seem in disagreement with previous reports that increased level of difficulty leads to longer sampling durations and/or decreased accuracy [2,31,32]. In these previous reports, difficulty was increased by asking mice or rats to distinguish more and more similar odorant mixtures at similar overall concentrations. In our case, the odor stayed the same but difficulty increased by lowering the odorant concentration. It is interesting to speculate if these two tasks present different types of difficulties to the animal, which it deals with differently by adopting different sniffing and sampling strategies.

The measurements we report were made with rigorous controls to exclude the use of sources of information available to the mice other than odor identity. As stated above, these extraneous cues include the noise of valve operation and vibrations associated with valve operation, among others. It also proved critical, particularly during assessment of the accuracy of odorant identity at low odor concentrations, to verify with further testing that on a given day the mouse was still motivated to reliably report odor identity when given post-session tests with a standard readily identified odorant concentration as a test of maintained odor identification ability.

Mice can perform trials at maximum speed at the cost of reduced accuracy of odor identification. A systematic study of speed-accuracy tradeoff by mice in the same olfactometer system used in the present study [31,35] showed that difficult odor discrimination tasks, e.g., discriminating a mixture of 60% odor A plus 40% odor B versus 40% odor A plus 60% odor B, could be reliably solved by mice but only if they were forced to take a more prolonged odor sampling time than the sampling time they would choose under free sampling conditions [31]. Since in the experiments reported here mice were only penalized for making an error with a longer inter-trial interval, some mice behave as though they are making decisions as fast as possible without regard to accuracy, hence the need for the control procedures implemented in this study.

Interestingly, when the mice decided to visit the water port after a nose poke in the odor port their transit times were significantly shorter for correct trials (Hits) versus incorrect trials (FAs). The transit time, typically 0.5–0.7 s (Fig 1C) has the character of a ballistic motor output that once triggered reflects only the execution of motor output, presumably, modulated by cognitive decision-making processes. In other contexts, this represents the transition from an evidence accumulation phase to a decision execution phase [36,37]. Given an estimate of the time required for decision execution and initiation of motor behavior by the mice, one or more of the later sniffs taken just prior to the termination of the nose poke in the odor port are unlikely to have contributed further evidence to the decision implemented by the mice.

In a second behavioral paradigm, we investigated olfactory performance and sniffing behavior in the presence of background odorants that were same as or different than the target odorant. Mice could maintain high accuracy in distinguishing the target propanol odor in the presence of a background of propanol odor until the highest (Nt) concentration, when accuracy dropped to near chance levels. Accuracy was not reduced in the presence of a dissimilar background odorant (eugenol), indicating that it was not the presence of a high odorant concentration itself, but more likely adaptation, either at the olfactory receptor neuron level or centrally in the olfactory bulb, to the target odorant that caused the decline in accuracy. Similar to the dose response experiments above, the sampling duration was longer for Hits when compared to CRs and FAs, again suggesting that mice sample for shorter duration in the absence of target or control odorants. Under the background paradigm, the transit time between the odor and the water port was also longer for FAs when compared to Hits (Fig 5E), suggesting that the execution phase in this case is also longer when an incorrect decision is made. One might speculate that this is due to the animal contemplating its decision or that it might already “know” that it will not receive a water reward and is therefore less motivated to seek out the water port. Our data might hint, but not unambiguously, that it is the former as for tasks performed at a low accuracy (e.g. 10^−6.5^ and MO in the dose response data and Nt in the adaptation data) the times between ports are more similar to port transit times for Hits and FAs.

The number of sniffs taken varied with the behavioral outcome exhibited by the mice with more sniffs being taken when mice scored Hits compared to CRs and FAs (Fig 6D). This raises the question if it is the behavioral outcome that determines the pattern of odorant sampling while in the odor port or the presence of the odorant and its sampling. Given that FAs (when no odor is present but the behavioral outcome is entering the water port) are more similar to CRs (absence of odorants), this suggests that sniffing patterns are indeed driven by odorant sampling.

In conclusion, our data suggest that mice can dynamically and task dependently modulate their odorant sampling strategies. Future work can address why mice chose to sample for longer and take more sniffs than necessary when exposed to odorants and what the benefit of such a strategy might be.

## References

[1] Resulaj A, Rinberg D (2015) Novel Behavioral Paradigm Reveals Lower Temporal Limits on Mouse Olfactory Decisions. Journal of Neuroscience 35: 11667–11673. 10.1523/JNEUROSCI.4693-14.2015 26290243 PMC4540801

[2] Uchida N, Mainen ZF (2003) Speed and accuracy of olfactory discrimination in the rat. Nature Neuroscience 6: 1224–1229. 10.1038/nn1142 14566341

[3] Uchida N, Kepecs A, Mainen ZF (2006) Seeing at a glance, smelling in a whiff: rapid forms of perceptual decision making. Nature reviews Neuroscience 7: 485–491. 10.1038/nrn1933 16715056

[4] Kepecs A, Uchida N, Mainen ZF (2007) Rapid and precise control of sniffing during olfactory discrimination in rats. Journal of Neurophysiology 98: 205–213. 10.1152/jn.00071.2007 17460109

[5] Laing DG (1983) Natural sniffing gives optimum odour perception for humans. Perception 12: 99–117. 10.1068/p120099 6657430

[6] Lefevre L, Courtiol E, Garcia S, Thevenet M, Messaoudi B, et al (2016) Significance of sniffing pattern during the acquisition of an olfactory discrimination task. Behavioural Brain Research 312: 341–354. 10.1016/j.bbr.2016.06.039 27343936

[7] Shusterman R, Smear MC, Koulakov AA, Rinberg D (2011) Precise olfactory responses tile the sniff cycle. Nat Neurosci 14: 1039–1044. 10.1038/nn.2877 21765422 PMC13348895

[8] Smear M, Shusterman R, O'Connor R, Bozza T, Rinberg D (2011) Perception of sniff phase in mouse olfaction. Nature 479: 397–400. 10.1038/nature10521 21993623

[9] Diaz-Quesada M, Youngstrom IA, Tsuno Y, Hansen KR, Economo MN, et al (2018) Inhalation Frequency Controls Reformatting of Mitral/Tufted Cell Odor Representations in the Olfactory Bulb. Journal of Neuroscience 38: 2189–2206. 10.1523/JNEUROSCI.0714-17.2018 29374137 PMC5830510

[10] Jordan R, Fukunaga I, Kollo M, Schaefer AT (2018) Active Sampling State Dynamically Enhances Olfactory Bulb Odor Representation. Neuron 98: 1214–1228 e1215. 10.1016/j.neuron.2018.05.016 29861286 PMC6030445

[11] Carey RM, Wachowiak M (2011) Effect of sniffing on the temporal structure of mitral/tufted cell output from the olfactory bulb. J Neurosci 31: 10615–10626. 10.1523/JNEUROSCI.1805-11.2011 21775605 PMC3159407

[12] Courtiol E, Hegoburu C, Litaudon P, Garcia S, Fourcaud-Trocme N, et al (2011) Individual and synergistic effects of sniffing frequency and flow rate on olfactory bulb activity. J Neurophysiol 106: 2813–2824. 10.1152/jn.00672.2011 21900510

[13] Verhagen JV, Wesson DW, Netoff TI, White JA, Wachowiak M (2007) Sniffing controls an adaptive filter of sensory input to the olfactory bulb. Nature Neuroscience 10: 631–639. 10.1038/nn1892 17450136

[14] Munch D, Galizia CG (2017) Take time: odor coding capacity across sensory neurons increases over time in Drosophila. Journal of comparative physiology A, Neuroethology, sensory, neural, and behavioral physiology 203: 959–972. 10.1007/s00359-017-1209-1 28852844 PMC5696509

[15] Kurnikova A, Moore JD, Liao SM, Deschenes M, Kleinfeld D (2017) Coordination of Orofacial Motor Actions into Exploratory Behavior by Rat. Current biology: CB 27: 688–696. 10.1016/j.cub.2017.01.013 28216320 PMC5653531

[16] Moore JD, Kleinfeld D, Wang F (2014) How the brainstem controls orofacial behaviors comprised of rhythmic actions. Trends in neurosciences 37: 370–380. 10.1016/j.tins.2014.05.001 24890196 PMC4100695

[17] McElvain LE, Friedman B, Karten HJ, Svoboda K, Wang F, et al (2018) Circuits in the rodent brainstem that control whisking in concert with other orofacial motor actions. Neuroscience 368: 152–170. 10.1016/j.neuroscience.2017.08.034 28843993 PMC5849401

[18] Kurnikova A, Deschenes M, Kleinfeld D (2019) Functional brain stem circuits for control of nose motion. Journal of neurophysiology 121: 205–217. 10.1152/jn.00608.2018 30461370 PMC6383659

[19] Grimaud J, Murthy VN (2018) How to monitor breathing in laboratory rodents: a review of the current methods. Journal of Neurophysiology 120: 624–632. 10.1152/jn.00708.2017 29790839 PMC6139454

[20] McAfee SS, Ogg MC, Ross JM, Liu Y, Fletcher ML, et al (2016) Minimally invasive highly precise monitoring of respiratory rhythm in the mouse using an epithelial temperature probe. Journal of neuroscience methods 263: 89–94. 10.1016/j.jneumeth.2016.02.007 26868731 PMC4801653

[21] Jordan R, Kollo M, Schaefer AT (2018) Sniffing Fast: Paradoxical Effects on Odor Concentration Discrimination at the Levels of Olfactory Bulb Output and Behavior. eNeuro 5. 10.1523/ENEURO.0148-18.2018 PMC6306510 30596145

[22] Short SM, Wachowiak M (2019) Temporal Dynamics of Inhalation-Linked Activity across Defined Subpopulations of Mouse Olfactory Bulb Neurons Imaged In Vivo. eNeuro 6. 10.1523/ENEURO.0189-19.2019 PMC6597857 31209151

[23] Losacco J, Ramirez-Gordillo D, Gilmer J, Restrepo D (2020) Learning improves decoding of odor identity with phase-referenced oscillations in the olfactory bulb. eLife 9. 10.7554/eLife.52583 PMC6986879 31990271

[24] Okun M (2020) Time Warping Reveals Hidden Features of Neuronal Population Responses. Neuron 105: 203–204. 10.1016/j.neuron.2019.12.010 31972140

[25] Williams AH, Poole B, Maheswaranathan N, Dhawale AK, Fisher T, et al (2020) Discovering Precise Temporal Patterns in Large-Scale Neural Recordings through Robust and Interpretable Time Warping. Neuron 105: 246–259 e248. 10.1016/j.neuron.2019.10.020 31786013 PMC7336835

[26] Reisert J, Golden GJ, Matsumura K, Smear M, Rinberg D, et al (2014) Comparing thoracic and intra-nasal pressure transients to monitor active odor sampling during odor-guided decision making in the mouse. Journal of Neuroscience Methods 221: 8–14. 10.1016/j.jneumeth.2013.09.006 24056232 PMC3858470

[27] Hess P, Clozel M, Clozel JP (1996) Telemetry monitoring of pulmonary arterial pressure in freely moving rats. Journal of Applied Physiology 81: 1027–1032. 10.1152/jappl.1996.81.2.1027 8872676

[28] Mills PA, Huetteman DA, Brockway BP, Zwiers LM, Gelsema AJ, et al (2000) A new method for measurement of blood pressure, heart rate, and activity in the mouse by radiotelemetry. Journal of Applied Physiology 88: 1537–1544. 10.1152/jappl.2000.88.5.1537 10797109

[29] Slotnick B, Restrepo D (2005) Olfactometry with mice Current Protocols in Neuroscience. Hoboken, NJ: Wiley InterScience. 10.1002/0471142301.ns0820s33 18428626

[30] Bodyak N, Slotnick B (1999) Performance of mice in an automated olfactometer: odor detection, discrimination and odor memory. Chemical Senses 24: 637–645. 10.1093/chemse/24.6.637 10587496

[31] Rinberg D, Koulakov A, Gelperin A (2006) Speed-accuracy tradeoff in olfaction. Neuron 51: 351–358. 10.1016/j.neuron.2006.07.013 16880129

[32] Abraham NM, Spors H, Carleton A, Margrie TW, Kuner T, et al (2004) Maintaining accuracy at the expense of speed: stimulus similarity defines odor discrimination time in mice. Neuron 44: 865–876. 10.1016/j.neuron.2004.11.017 15572116

[33] Kelliher KR, Ziesmann J, Munger SD, Reed RR, Zufall F (2003) Importance of the CNGA4 channel gene for odor discrimination and adaptation in behaving mice. Proceedings of the National Academy of Sciences of the United States of America 100: 4299–4304. 10.1073/pnas.0736071100 12649326 PMC153087

[34] Kepecs A, Uchida N, Mainen ZF (2006) The sniff as a unit of olfactory processing. Chemical Senses 31: 167–179. 10.1093/chemse/bjj016 16339265

[35] Rinberg D, Koulakov A, Gelperin A (2006) Sparse odor coding in awake behaving mice. Journal of Neuroscience 26: 8857–8865. 10.1523/JNEUROSCI.0884-06.2006 16928875 PMC6674368

[36] Tsetsos K, Pfeffer T, Jentgens P, Donner TH (2015) Action Planning and the Timescale of Evidence Accumulation. PloS ONE 10: e0129473 10.1371/journal.pone.0129473 26068458 PMC4467085

[37] Piet AT, El Hady A, Brody CD (2018) Rats adopt the optimal timescale for evidence integration in a dynamic environment. Nature Communications 9: 4265 10.1038/s41467-018-06561-y 30323280 PMC6189050

